# Non-Assisted Hatching Trophectoderm Biopsy Does Not Increase The Risks of Most Adverse Maternal and Neonatal Outcome and May Be More Practical for Busy Clinics: Evidence From China

**DOI:** 10.3389/fendo.2022.819963

**Published:** 2022-02-17

**Authors:** Shuo Li, Shuiying Ma, Jialin Zhao, Jingmei Hu, Hongchang Li, Yueting Zhu, Wenjie Jiang, Linlin Cui, Junhao Yan, Zi-Jiang Chen

**Affiliations:** ^1^ Center for Reproductive Medicine, Cheeloo College of Medicine, Shandong University, Jinan, China; ^2^ Key Laboratory of Reproductive Endocrinology of Ministry of Education, Shandong University, Jinan, China; ^3^ Shandong Key Laboratory of Reproductive Medicine, Shandong University, Jinan, China; ^4^ Shandong Provincial Clinical Research Center for Reproductive Health, Shandong University, Jinan, China; ^5^ National Research Center for Assisted Reproductive Technology and Reproductive Genetics, Shandong University, Jinan, China; ^6^ Shanghai Key Laboratory for Assisted Reproduction and Reproductive Genetics, Ren Ji Hospital, School of Medicine, Shanghai Jiao Tong University, Shanghai, China; ^7^ Center for Reproductive Medicine, Ren Ji Hospital, School of Medicine, Shanghai Jiao Tong University, Shanghai, China

**Keywords:** elective single-embryo transfer, gestational diabetes mellitus, non-assisted hatching trophectoderm biopsy, perinatal outcomes, preimplantation genetic testing

## Abstract

**Objective:**

This study was conducted in order to investigate whether non-assisted hatching trophectoderm (TE) biopsy increases the risks of adverse perinatal outcomes in livebirths following elective single cryopreserved-thawed blastocyst transfer.

**Patients and Methods:**

A total of 5,412 cycles from 4,908 women who achieved singleton livebirths between 2013 and 2019 were included in this retrospective cohort study. All embryos in this study were fertilized by intracytoplasmic sperm injection (ICSI) and cryopreserved through vitrification. The main intervention is to open the zona pellucida (ZP) of day 5/6 blastocyst immediately for biopsy without pre-assisted hatching. The main outcome measures are the common maternal and neonatal outcomes, including hypertensive disorders of pregnancy (HDPs), gestational diabetes mellitus (GDM), abnormal placentation, abnormalities in umbilical cord and amniotic fluid, preterm birth, cesarean section, low birth weight, postpartum hemorrhage, and prolonged hospital stay (both mothers and infants). The generalized estimation equation (GEE) was used to control the effects of repeated measurements. The non-conditional logistic regression model was used to examine the associations between embryo biopsy status and each adverse perinatal event. Given that the selection bias and changes in learning curve might affect the results, we selected 1,086 similar (matching tolerance = 0.01) cycles from the ICSI group *via* propensity score matching (PSM) for second comparisons and adjustment (conditional logistic regression).

**Results:**

After adjusting for confounders, we confirmed that the non-assisted hatching protocol did not increase the risks of most adverse maternal and neonatal outcomes. Despite this, there were increased risks of GDM (aOR: 1.522, 95% CI: 1.141–2.031) and umbilical cord abnormalities (aOR: 11.539, 95% CI: 1.199–111.067) in the biopsy group. In the second comparisons after PSM, GDM incidence in the biopsy group was still higher (7.26% vs. 5.16%, *P* = 0.042), yet all measurement outcomes were equally likely to occur in both groups after the second adjustment.

**Conclusions:**

The non-assisted hatching TE biopsy does not increase the risks of most adverse perinatal outcomes. However, there is a higher GDM incidence in the biopsy group, and this association warrants further study. Considering its safety and simplicity, the non-assisted hatching protocol has the potential to become the preferred option for TE biopsy, especially in busy clinics and IVF laboratories.

## Introduction

Preimplantation genetic testing (PGT) is now a widely used tool, and its share of total assisted reproductive technology (ART) cycles nearly doubled in the USA and UK from 2014 to 2017 (1). Possible drivers of such use include the preference to have children after the age of 35, the clinical promotion of new sequencing techniques, the need to block hereditary diseases, and the rising number of unexplained infertilities. Compared with *in-vitro* fertilization (IVF) and intracytoplasmic sperm injection (ICSI), embryo biopsy is the main technique in the PGT procedure and the most intrusive intervention for the embryo. The method to obtain 4–10 cells from the ectoderm of the blastocyst (5–7 days after fertilization) was defined as trophectoderm (TE) biopsy (2), which significantly reduced the proportion of biopsied cells in the total cell count of an embryo. Due to the little influence on developmental potential and high diagnostic accuracy (3), this gradually replaced the previous polar body (PB) biopsy and blastomere biopsy, becoming the mainstream approach in most centers.

Currently, there are three main strategies for TE biopsy: the day 3 and day 4 hatching-based strategies, the same-day hatching-based strategy, and the simultaneous zona pellucida (ZP) opening and TE cell retrieval strategy (4). The main difference resides on whether the ZP needs to be opened for pre-assisted hatching. The first two require a pre-drilled hole of about 5 µm in the ZP of cleavage/morula/blastula-stage embryos to allow TE cells to herniate for biopsy. The last one, the ZP of day 5/6/7 blastocyst, is open instantly before the biopsy; thus, it was also called the “day 5–7 sequential ZP opening and TE cells retrieval approach” (4). Protocols that require assisted hatching present several issues such as the risk of the early gap thinning the ZP and affecting the normal expansion of blastocyst (5) as it has been reported that laser opening at cleavage might reduce the development potential (6) and alter epigenetic modification (7) of embryos. Additionally, the component of the blastocyst cells that were hatched out might be uncontrollable, and if hatching starts from the inner cell mass (ICM), biopsy could only be performed once the blastocyst had completely hatched out. During this period, the embryos need to be observed more frequently, which not only prolonged the *in-vitro* exposure time of the embryos but also significantly increased the workloads of the embryologist. Finally, the timing required for hatching out is uncertain and the development speed for each embryo is not exactly in sync, requiring the operators to biopsy these embryos in batches. By contrast, the protocol without pre-assisted hatching does not require assisted laser drilling in advance; thus, the embryo can remain undisturbed until the blastocyst stage. Meanwhile, the blastocysts with different characteristics can be handled flexibly and the ICM can be clearly identified during the drilling and aspirating process. It is generally better to vitrify a collapsed blastocyst, and non-assisted hatching protocol exactly has the characteristic of easily inducing embryo collapse. Some reports suggest that cycles adopting the non-assisted hatching protocol achieved better pregnancy outcomes [blastocyst frozen rate (8), thawing survival rate/clinical pregnancy rate (9), and lower mosaic rate (10)], but some uncertainty remains regarding non-assisted hatching biopsy. Hatching-based protocols only require retrieval of cellular components outside the ZP leaving other embryonic components in the ZP less affected, particularly the ICM. Contrastingly, the pulling and aspiration movements are more intense during the non-assisted hatching biopsy, and the manipulations must be adapted to the characteristics of each blastocyst, sometimes requiring extra steps such as injection of culture medium (11).

The debate over the benefits and risks of PGT has persisted. Some studies reported that embryo biopsy did not increase the risks related to abnormal placentation, maternal complications, and neonatal adverse events (12–16). However, others claimed that embryo biopsy increased the incidence of pregnancy-induced hypertension (PIH) or preeclampsia (17, 18), preterm birth (19), and small-term for gestational age of the baby (20). The differences in biopsied time, position, and method might explain the inconsistency of these observations. Moreover, some IVF pregnancies were also mixed in the control group of previous studies. Since the fertilization method was different from that of the PGT group, we believe the conclusions of these studies are still open to discussion. Some embryologists have proposed that the TE biopsy protocols can affect pregnancy outcomes (9, 10), but in previous studies made by clinicians, they either adopted based day-3 hatching protocol or did not control for protocol types.

The invasive activity and syncytial degree of TE during the early stages of pregnancy directly determined the structure and function of mature placenta in late pregnancy. Considering that abnormal placentation could lead to a series of adverse perinatal events and higher long-term risks of chronic diseases in women and their offspring with obstetric complication histories (21), it is necessary to clarify the association between TE biopsy and each adverse perinatal event. Our goal is to provide definitive evidence with a large sample size to assess the safety of non-assisted hatching TE biopsy and PGT.

## Material and Methods

### Study Populations

**Figure 1:**
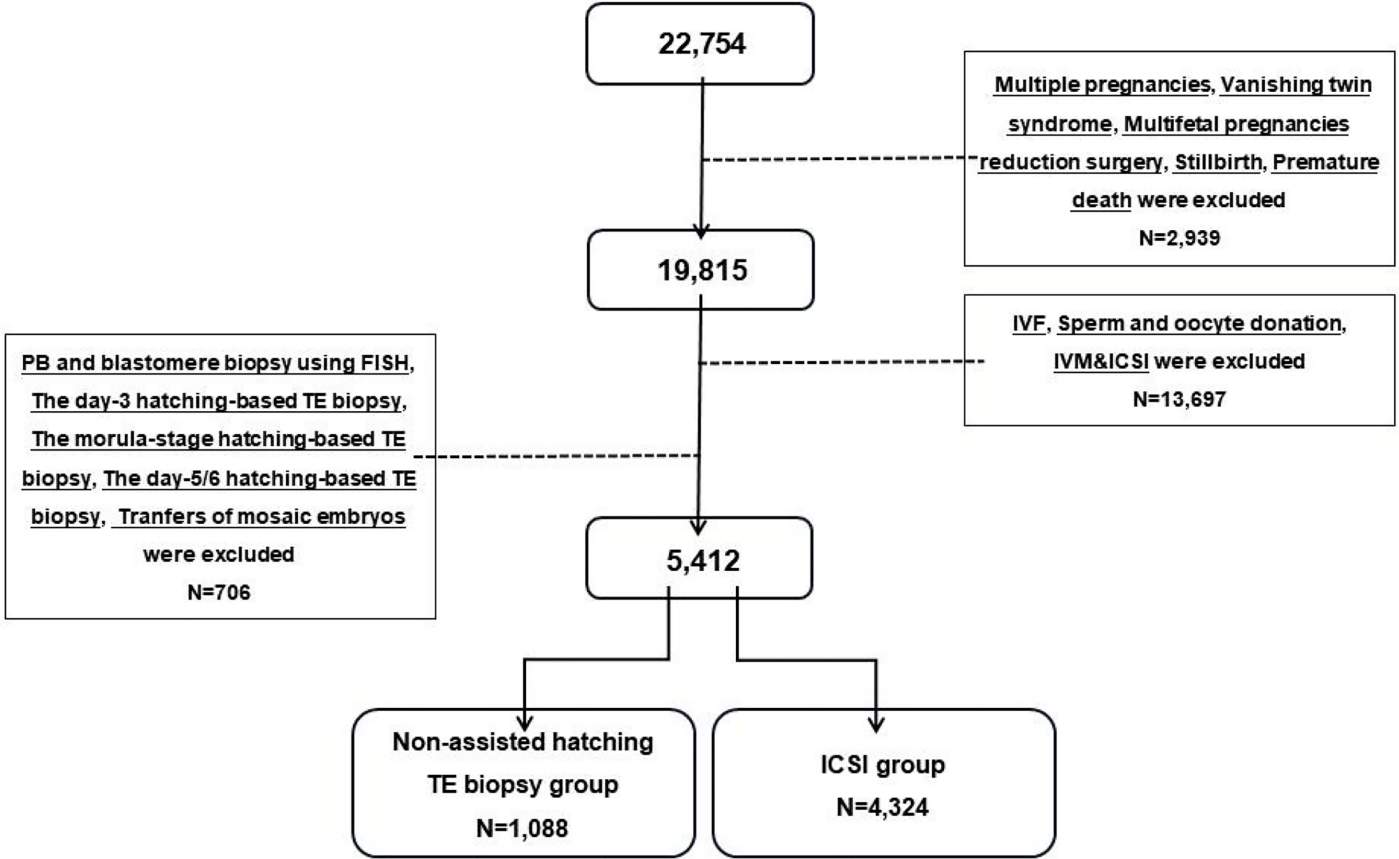
Flowchart of the study populations. IVF, *in-vitro* fertilization; ICSI, intracytoplasmic sperm injection; IVM, *in-vitro* maturation; PB, polar body; TE, trophectoderm.

A total of 22,754 cryopreserved-thawed cycles that achieved livebirths at the Center for Reproductive Medicine, Shandong University from 2011 to 2019 were included in our screening range. After excluding IVF cycles, transfers of mosaic embryo, multiple pregnancies, PB and blastomere biopsy, hatching-based TE biopsy, and others (stillbirth, premature death, vanishing twin syndrome, multifetal pregnancy reduction surgery), 5,412 cycles from 4,908 women were included in this retrospective study (a detailed flowchart is shown in **Figure 1**). The subjects in the ICSI group (*n* = 4,324) were transferred to a vitrified cryopreserved-thawed blastocyst fertilized by ICSI from 2013 to 2018. The subjects in the biopsy group (*n* = 1,088) received a biopsied blastocyst following general PGT procedure from 2014 to 2019. To keep the fertilization method consistent, the couples in the ICSI group underwent ART mainly due to male factor infertility, which might cause the basic physical conditions of women in the ICSI group to be better than those of the biopsy group. The non-assisted hatching TE biopsy was used for all transfer cycles in the biopsy group, which eliminated the interference of biopsy protocols.

This study was approved by the Ethics Committee of Reproductive Medicine Center of Shandong University, and all participants consented to their information being used for scientific research anonymously.

### PGT Procedure

**Figure 2:**
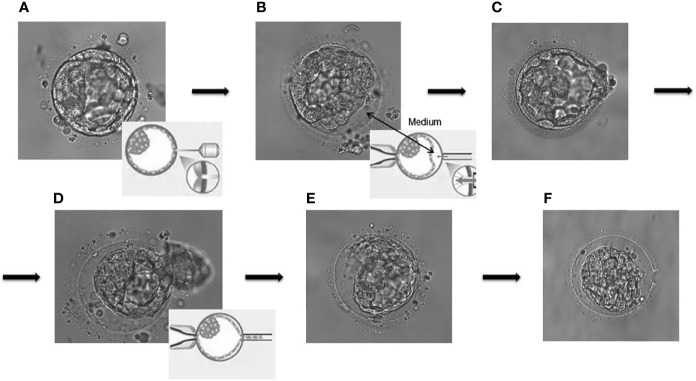
The common procedure for non-assisted hatching trophectoderm biopsy. **(A)** Laser drilling; **(B)** artificial separation by injection of culture medium; **(C, D)** pulling and suction; **(E, F)** the remaining blastocyst components collapsed in the zone pellucida.

Controlled ovarian hyperstimulation (COH) was performed according to the clinical routines of our center, monitoring serum sex hormones and ultrasonography, and the dosage of gonadotropins was timely adjusted based on the response of the ovary. When at least two follicles reached 18 mm in diameter, exogenous recombinant human chorionic gonadotropin (rhCG) was injected and the oocytes were retrieved 24–36 h later. Oocytes that develop to metaphase II (MII) were fertilized by ICSI as previously described (8). Zygotes were cultured *in vitro* to the blastocyst stage (5–7 days) and cryopreserved through vitrification using the Mukaida protocol with cryoloop (22). For the embryos requiring PGT, the ZP was opened immediately and 4–10 TE cells were removed from high-quality blastocysts (>4BC) (23) through laser-mediated drilling (RI, England, Saturn Active) before freezing. TE cells were then rinsed three times in 1% polyvinyl pyrrolidone (PVP, Scandinavian IVF Science, Sweden, 10111) and enclosed into PCR tubes containing 2 µl phosphate-buffered saline (PBS, Solarbio, USA, P1020). The concrete experimental operation was conducted by equally skilled embryologists and the blastocysts were scored in accordance with the Gardner standards (24). **Figure 2** illustrates the common procedure for non-assisted hatching TE biopsy. DNA was extracted from the isolated blastula cells and amplified (SurePlex whole genome amplification kit, Illumina, San Diego, CA, USA) to meet the sample requirements for the subsequent next-generation sequencing (NGS) or comparative genomic hybridization (CGH). The genetic diagnoses were made by a panel of professional geneticists.

At the second (or later) menstrual cycles after oocyte retrieval, endometrial preparation was performed, and the clinicians decided on the most appropriate implantation protocol based on the endometrial status of the patients and the previous embryo transfer history. Only one qualified blastocyst was selected for transplantation based on Gardner scoring and the advice of geneticists. The data of maternal and neonatal outcomes were collected 42 days after childbirth through clinical medical records and telephone follow-up.

### Diagnostic Criteria

The diagnosis of polycystic ovarian syndrome (PCOS) was based on the consensus issued by the European Society of Human Reproduction and Embryology (ESHRE)/American Society for Reproductive Medicine (ASRM) in 2004 (25) and the Chinese Ministry of Health in 2011 (http://hbba.sacinfo.org.cn/stdDetail/78020832ca41940e1d0665507a75b539). The diagnoses of diabetes were based on the standard released by the American Diabetes Association (ADA) in 2010 (26). Thyroid disorders involved in this study included hyperthyroidism, hypothyroidism, chronic thyroiditis, and history of surgery or iodine treatment, the diagnoses of which were referred to the guideline recommended by the American Thyroid Association (ATA) in 2011 (27).

The diagnoses of HDPs were based on the guidelines issued by the American College of Obstetricians and Gynecologists (ACOG) in 2013 (28) and the Chinese Ministry of Health in 2012 (http://hbba.sacinfo.org.cn/stdDetail/d74604a6950738b4faf7e9ee34aa7b99). The diagnosis of gestational diabetes mellitus (GDM) was based on the criteria published by the International Association of Diabetes and Pregnancy Study Groups (IADPSG) in 2010 (29) and the Chinese Ministry of Health in 2011 (http://hbba.sacinfo.org.cn/stdDetail/97f630da575d4db3e9eee2e6ca3d1f45).

Other covariates were defined as follows: uterine congenital anomalies (infantile, unicornous, rudimentary horn, didelphic, bicornuate, arcuate), abnormal placentation (abruption, previa, increta, percreta, accreta), umbilical cord abnormalities (knot, torsion, polyp, vessel malformation), abnormal amniotic fluid (oligohydramnios, polyhydramnios, 3-degree contamination), preterm birth (<37 weeks), low birth weight (<2,500 g), postpartum hemorrhage (>500 ml for vaginal delivery or >1,000 ml for cesarean section), prolonged stay for mothers (>3 days for vaginal delivery or >5 days for cesarean section), and prolonged stay for infants (>3 days for vaginal delivery or >5 days for cesarean section among children >35 weeks of gestational age).

### Statistical Analyses

To avoid the influence of blood sampling time, the values of hCG were transformed to hCG ratio (serum hCG concentration/gestational day at sampling). Because one woman might contribute to multiple transfer cycles in this study, a generalized estimation equation (GEE) was used to control the effects of repeated measurements. In GEE, the adjusted odds ratios (aORs) and 95% confidence intervals (CIs) derived from the non-conditional logistic regression model were used to describe the associations between embryo biopsy status and each adverse perinatal event. Considering the selection bias and the changes in learning curve might impact the results, we selected 1,086 similar (matching tolerance: 0.01) cycles from the ICSI group through propensity score matching (PSM) for second comparisons. The predictive variables included the maternal birth date, maternal age, maternal BMI, date of embryo biopsy, date of embryo transfer, date of delivery, parity, neonatal weight, and neonatal sex. A conditional logistic regression model was used for the second adjustment of the matched data.

Statistical tests were two-sided and *P*-value <0.05 was considered statistically significant. Due to the low percentage (<2%) of missing data, we supplemented the missing data through mean imputation. All analyses were conducted with software SPSS (version 25.0, Chicago, IL, USA).

## Results

**Table 1** summarizes the demographic characteristics of subjects in the two groups. There were statistical differences in age (31 vs. 30, *P* < 0.001), BMI (22.66 vs. 22.27, *P* < 0.001), times of previous miscarriages (*P* < 0.001), parity (*P* = 0.026), endometrial preparation protocols (*P* < 0.001), endometrial thickness of transfer day (0.9 vs. 1.0, *P* < 0.001), and hCG ratio (56.36 vs. 60.53, *P* < 0.001) between the groups. As for maternal basic diseases, there were statistical differences in thyroid disorders (11.76% vs. 14.43%, *P* = 0.023), chronic hypertension (1.29% vs. 2.43%, *P* = 0.022), family history of hypertension (15.81% vs. 11.22%, *P* < 0.001), family history of diabetes (6.71% vs. 4.35%, *P* = 0.001), and history of uterine surgery (13.05% vs. 16.93%, *P* = 0.002) between the groups.

**Table 1:** Comparisons of demographic characteristics between the non-assisted hatching biopsy group and the ICSI group.

	Non-assisted hatching biopsy group (*n* = 1,088)	ICSI group (*n* = 4,324)	*P*-value
Age (years)*	31 (3.5)	30 (3)	<0.001^a^
BMI (kg/m²)*	22.66 (2.23)	22.27 (2.39)	0.004^a^
Times of previous miscarriages*	None: 32.81% (357)	None: 76.48% (3,307)	<0.001^b^
	Once: 19.67% (214)	Once: 18.94% (819)	
	Twice or more: 47.52% (517)	Twice or more: 4.58% (198)	
Parity*	Primiparous: 72.24% (786)	Primiparous:75.51% (3,265)	0.026^b^
	Multiparous: 27.76% (302)	Multiparous: 24.49% (1,059)	
Endometrial preparation protocols*	NC: 49.63% (540)	NC: 58.00% (2,508)	<0.001^b^
	HRT: 37.04% (403)	HRT: 31.06% (1,343)	
	OI: 11.58% (126)	OI: 9.53% (412)	
	Others: 1.75% (19)	Others: 1.41% (61)	
Endometrial thickness of transfer day (cm)*	0.9 (0.1)	1.0 (0.1)	<0.001^a^
Serum hCG level (IU/L)/gestational day at sampling (days)*	56.36 (23.71)	60.53 (24.22)	<0.001^a^
PCOS	16.64% (181)	17.55% (759)	0.475^b^
Uterine congenital anomalies	2.21% (24)	1.50% (65)	0.103^b^
Untreated uterine fibroid	3.49% (38)	3.45% (149)	0.940^b^
Thyroid disorders*	11.76% (128)	14.43% (624)	0.023^b^
Chronic hypertension*	1.29% (14)	2.43% (105)	0.022^b^
Family history of hypertension*	15.81% (172)	11.22% (485)	<0.001^b^
Type 1 or type 2 diabetes	3.68% (40)	2.59% (112)	0.053^b^
Family history of diabetes*	6.71% (73)	4.35% (188)	0.001^b^
History of uterine surgery*	13.05% (142)	16.93% (732)	0.002^b^

Data are shown as median (quartile deviation, QD) and (%) (number of positive cases).

ICSI, intracytoplasmic sperm injection; BMI, body mass index; NC, nature cycle; HRT, hormone replacement treatment; OI, ovulation induction; hCG, human chorionic gonadotropin; PCOS, polycystic ovarian syndrome.

a Mann–Whitney U test.

b Pearson’s chi-squared test. *Statistically significant.

Comparisons of maternal and neonatal outcomes are shown in **Table 2**. Women in the biopsy group had a higher incidence of GDM (7.26% vs. 5.04%, *P* = 0.004) than those in the ICSI group. There were no statistical differences in PIH and preeclampsia (*P* = 0.784), preeclampsia with severe features and eclampsia (*P* = 0.517), HDP with GDM (*P* = 0.608), abnormal placentation (*P* = 0.341), umbilical cord abnormalities (*P* = 0.059), abnormal amniotic fluid (*P* = 0.804), preterm birth (*P* = 0.809), delivery mode (*P* = 0.782), neonatal sex (*P* = 0.099), low birth weight (*P* = 0.372), postpartum hemorrhage (*P* = 0.310), and prolonged stay for mothers (*P* = 0.188) and infants (*P* = 0.103).

**Table 2:** Comparisons of maternal and neonatal outcomes between the non-assisted hatching biopsy group and the ICSI group.

	Non-assisted hatching biopsy group (*n* = 1,088)	ICSI group (*n* = 4,324)	*P*-value
PIH + preeclampsia	4.96% (54)	4.76% (206)	0.784^a^
Preeclampsia with severe features + eclampsia	0.64% (7)	0.49% (21)	0.517^a^
GDM*	7.26% (79)	5.04% (218)	0.004^a^
HDP + GDM	0.55% (6)	0.42% (18)	0.608^b^
Abnormal placentation	1.38% (15)	1.04% (45)	0.341^a^
Umbilical cord abnormalities	0.28% (3)	0.05% (2)	0.059^b^
Abnormal amniotic fluid	1.10% (12)	1.02% (44)	0.804^a^
Preterm birth	6.43% (70)	6.64% (287)	0.809^a^
Delivery mode	Vaginal: 28.03% (305)	Vaginal: 27.61% (1,194)	0.782^a^
	Abdominal: 71.97% (783)	Abdominal: 72.39% (3,130)	
Neonatal sex ratio	Female: 46.78% (509)	Female: 49.58% (2,144)	0.099^a^
	Male: 53.22% (579)	Male: 50.42% (2,180)	
Low birth weight	4.50% (49)	3.91% (169)	0.372^a^
Postpartum hemorrhage	0.37% (4)	0.21% (9)	0.310^b^
Prolonged stay for mothers	1.93% (21)	1.39% (60)	0.188^a^
Prolonged stay for infants	5.61% (61)	4.44% (192)	0.103^a^

Data are shown as (%) (number of positive cases).

ICSI, intracytoplasmic sperm injection; PIH, pregnancy-induced hypertension; GDM, gestational diabetes; HDP, hypertensive disorders of pregnancy.

a Pearson’s chi-squared test.

b Fisher’s exact test. *Statistically significant.

**Figure 3:**
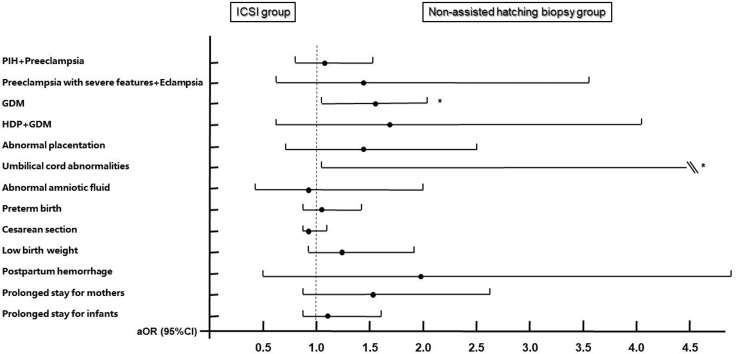
Adjusted odds ratios and 95% confidence intervals for maternal and neonatal outcomes by embryo biopsy status. Adjusted for maternal age, maternal BMI, times of previous miscarriages, parity, endometrial preparation protocols, endometrial thickness of transfer day, hCG ratio, PCOS, thyroid disorders, chronic hypertension, family history of hypertension, diabetes, family history of diabetes, history of uterine surgery, and neonatal sex. ICSI group is the reference group. PIH, pregnancy-induced hypertension; GDM, gestational diabetes; HDP, hypertensive disorders of pregnancy. * Statistically significant.

Increased risks of GDM (aOR: 1.522, 95% CI: 1.414–2.031, *P* = 0.004) and umbilical cord abnormalities (aOR: 11.539, 95% CI: 1.199–111.067, *P* = 0.034) were observed in the biopsy group when using the ICSI group as the reference after adjusting for confounding factors such as maternal age, maternal BMI, parity, times of previous miscarriages, endometrial preparation protocols, endometrial thickness of transfer day, hCG ratio, PCOS, thyroid disorders, chronic hypertension, family history of hypertension, diabetes, family history of diabetes, history of uterine surgery, and neonatal sex. The remaining 11 outcome variables did not show increased risks (**Figure 3**, see **Supplementary Table 1** for details).

**Figure 4:**
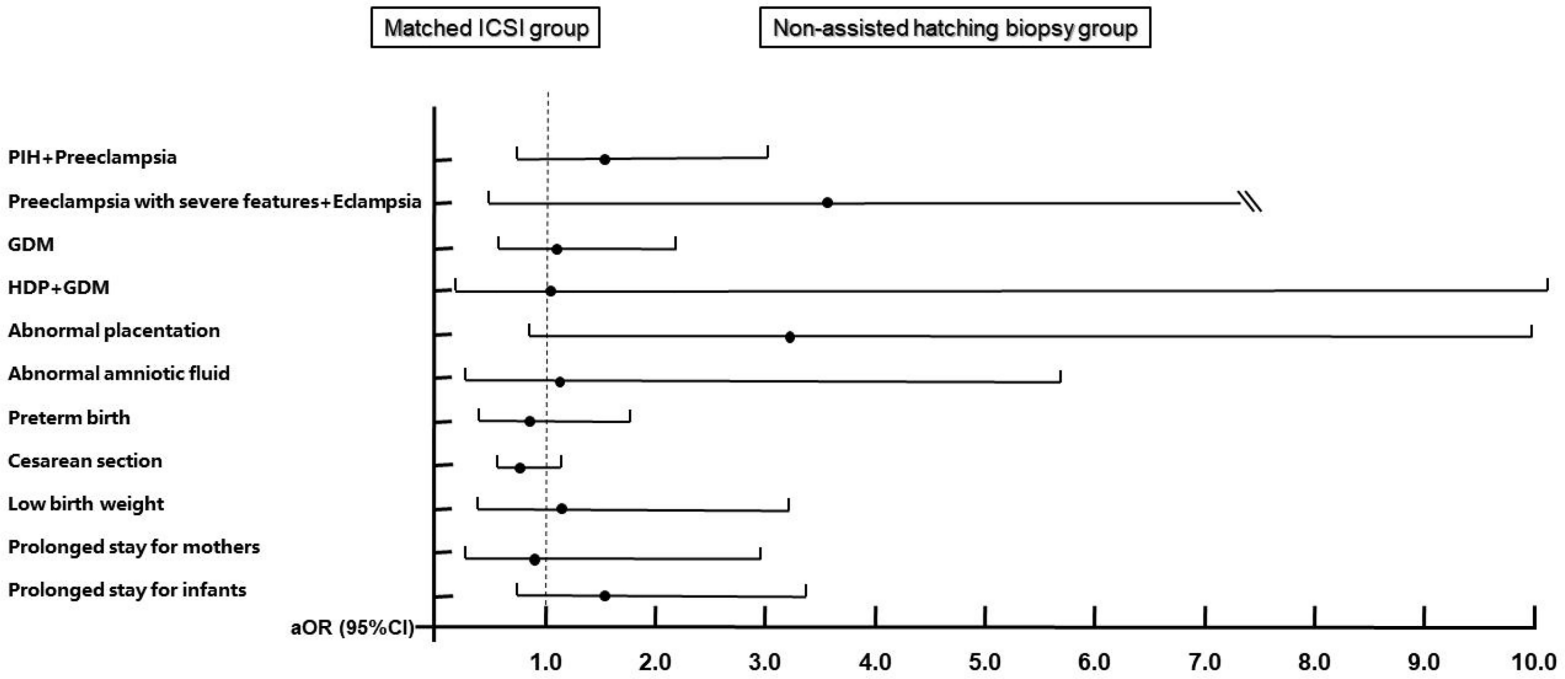
Adjusted odds ratios and 95% confidence intervals for maternal and neonatal outcomes after matching. Adjusted for maternal age, maternal BMI, times of previous miscarriages, parity, endometrial preparation protocols, endometrial thickness of transfer day, hCG ratio, PCOS, thyroid disorders, chronic hypertension, family history of hypertension, diabetes, family history of diabetes, and history of uterine surgery. Matched ICSI group is the reference group. PIH, pregnancy-induced hypertension; GDM, gestational diabetes; HDP, hypertensive disorders of pregnancy.

After PSM, the results of the second comparisons are shown in **Table 3**. Because the positive cases of umbilical cord abnormalities and postpartum hemorrhage in the matched ICSI group were zero, we were unable to compare and adjust these two outcome variables. Compared with the counterparts in the matched ICSI group, there were still more women with GDM in the biopsy group (7.26% vs. 5.16%, *P* = 0.042). **Figure 4** (details are available in **Supplementary Table 3**) displays the results from the conditional logistic regression model that none of the 11 perinatal outcomes in the biopsy group showed an additional risk.

**Table 3:** Comparisons of maternal and neonatal outcomes between the non-assisted hatching biopsy group and the matched ICSI group.

	Non-assisted hatching biopsy group (*n* = 1,088)	Matched ICSI group (*n* = 1,086)	*P*-value
PIH + preeclampsia	4.96% (54)	4.88% (53)	0.929^a^
Preeclampsia with severe features + eclampsia	0.64% (7)	0.55% (6)	0.783^a^
GDM*	7.26% (79)	5.16% (56)	0.042^a^
HDP + GDM	0.55% (6)	0.37% (4)	0.753^b^
Abnormal placentation	1.38% (15)	1.29% (14)	0.856^a^
Umbilical cord abnormalities	0.28% (3)	0% (0)	^–^
Abnormal amniotic fluid	1.10% (12)	0.64% (7)	0.251^a^
Preterm birth	6.43% (70)	6.54% (71)	0.922^a^
Delivery mode	Vaginal: 28.03% (305)	Vaginal: 24.59% (267)	0.068^a^
	Abdominal: 71.97% (783)	Abdominal: 75.41% (819)	
Neonatal sex ratio	Female: 46.78% (509)	Female: 43.46% (472)	0.120^a^
	Male: 53.22% (579)	Male: 56.54% (614)	
Low birth weight	4.50% (49)	3.22% (35)	0.121^a^
Postpartum hemorrhage	0.37% (4)	0% (0)	^–^
Prolonged stay for mothers	1.93% (21)	1.20% (13)	0.168^a^
Prolonged stay for infants	5.61% (61)	3.96% (43)	0.072^a^

Data are shown as (%) (number of positive cases).

ICSI, intracytoplasmic sperm injection; PIH, pregnancy-induced hypertension; GDM, gestational diabetes; HDP, hypertensive disorders of pregnancy.

a Pearson’s chi-squared test.

b Fisher’s exact test. *Statistically significant.

## Discussion

Our study demonstrates the safety of non-assisted hatching TE biopsy and provides a reference for embryologists when selecting protocols for TE biopsy. Due to its simplicity and fewer effects on embryo development potential, non-assisted hatching TE biopsy might be a better option for the busy IVF laboratories with large PGT cycles or countries with large populations.

Our conclusions are consistent with that of Swanson and her colleagues (30). In their previous study, PGT was not associated with the risks of most perinatal adverse events but GDM. However, this was based on IVF pregnancies in the control group and neglected the effects of TE biopsy protocols. With a much larger sample size and more controlled and homogeneous groups, our study reinforced these findings.

Except for the GDM incidence, our results are in general agreement with the conclusions by Sites et al. (16). They aggregated the perinatal data of ART cycles at multiple clinics through the state health system and concluded that embryo biopsy for PGT did not increase the odds for diagnoses related to abnormal placentation, maternal complications (including GDM), and prolonged stay (both mothers and infants). Similar to our study, to allow the roles of biopsy to be more clearly apparent, only singleton livebirths were included in their study, but some IVF cycles were also incorporated in their control group, and there was no adjustment for fertilization methods and biopsy protocol types. The applicable indications of PGT in east USA and the selection for biopsy protocols between different IVF laboratories might explain the difference in our observations. Moreover, our study included the data of endometrial preparations, a significant limitation they mentioned in their study. Another meta-analysis (31) reported 785,445 participants from different countries enrolled over an 11-year period and found a lower rate of “very low birth weight” and “cesarean section” and a higher rate of “preterm birth” and “intrauterine growth retardation” in PGT pregnancies, compared with those of IVF/ICSI pregnancies. Over such a longtime period and with such a large sample size, some associations can simply be due to chance. Non-standardization of experimental procedures and changes in learning curve could both impact the final conclusions. The differences in race and region might also be another confounding factor.

Some scholars advocated that PGT was associated with the risk of preeclampsia. Zhang et al. found that PGT increased the risk of preeclampsia (17); however, their report was based on a small sample size and included multiple pregnancies that might account for the discrepancy with our report. Makhijani et al. (18) also only included singleton births, but they adjusted the number of embryos transferred as a confounding factor, suggesting the cycles in their study were not all elective single-embryo transfers (eSET). Additionally, the biopsy protocols between us differed, and they adopted the day 3 hatching-based TE biopsy. A randomized controlled trial (RCT) has already shown that frozen-thawed embryo transfers had higher risks of preeclampsia (32). Considering the embryos in the PGT cycle are almost frozen-thawed, it is necessary to clarify whether the increased risk of preeclampsia is caused by freeze-thaw or biopsy. A reasonable physiological explanation for increased preeclampsia risk was not described in previous studies. Sunkara et al. (14) and Li et al. (19) concluded that PGT was not associated with adverse neonatal outcomes (though Sunkara found a slightly higher risk of preterm birth in the PGT group), which were basically consistent with our findings with neonates.

By comparison, each of the three biopsy strategies has its own advantages and disadvantages. However, limited by several disadvantages we mentioned in the *Introduction*, the hatching-based strategies are not particularly practical for some large clinics or IVF labs (2), especially in countries with a large population such as China or India. The changes in the population policy of the Chinese government increased the pressure over IVF laboratories with a large number of cycles, and many laboratories have gradually abandoned the hatching-based protocols which are more time-consuming and require a constant check. Nonetheless, this is only the choice under the stress of workloads, not based on evidence. As Rubino et al. (9) proposed, it is high time to focus on the blastocyst biopsy protocols. Because clinicians are not the ones performing the biopsy, the impact of different protocols for TE biopsy on the clinical outcomes is often ignored in their previous studies. Similarly, the evidence provided by embryologists focuses mostly on embryo quality and laboratory parameters, rarely involving maternal and neonatal conditions throughout the perinatal period. Because the biopsy protocol was well controlled, our research makes a powerful complement to previous observations.

In terms of the increased risk of GDM, we believe it could be attributed to many reasons. Lower hCG ratio in the biopsy group at the first gestational month might play an important part in the development of GDM (33). hCG can lead to thyroid-stimulating hormone (TSH) activity and induce free thyroxine (FT4) surge (34, 35), facilitate early placentation to indirectly affect insulin resistance (IR) derived by placental endocrine (36), and play as an immune modulator to alleviate pancreatic autoimmunity (37). Liu et al. (38) demonstrated that higher hCG levels in early pregnancy were associated with a lower risk of GDM and maternal FT4 which may act as an important mediator (24%) in this association. While some scholars (39, 40) claim that TE biopsy might reduce the level of serum beta-hCG in early pregnancies, this is still controversial (41). In our study, hCG values were measured within 1 month of embryo transfer; at a stage when serum hCG concentration doubles rapidly, we were unable to convert the concentration values into the median of multiples (MoMs) and perform further mediation analysis. Furthermore, it was not clear whether this decrease in hCG levels occurred only in the first month of pregnancy or continued into the first trimester. Future studies will collect longitudinal serum samples for hCG, FT4, and TSH measurement, to assess the difference between the biopsy group and the no-biopsy group. Secondly, considering chromosomal abnormality is one of the indications for PGT, we thought the couples in the biopsy group had more complex genetic backgrounds, such as translocation or inversion, than their counterparts in the ICSI group. Numerous single nucleotide polymorphisms (SNPs) in susceptibility genes associated with both glucose metabolism and placental development, such as ADIPOQ (42), IL1B (43, 44), and ABCC8 (45), might go undetected due to the absence of carrier screening (20). Thirdly, the percentage of Asian women (46) included in our study might also contribute to some discrepancies with previous studies. The risk of GDM is significantly higher in Asian women, whose BMI was lower than that of women in the general population. In contrast, these were the least likely ethnic group to receive recommended diabetes screening (47–49). In fact, this might explain why our results align with the study of Swanson et al. (30) which also included a high percentage of Asians.

However, our study presents several limitations. The particularity of PGT populations leads to significant demographic differences which cannot be controlled outside of an RCT. Besides, some data of perinatal outcomes were obtained through telephone follow-up, which inevitably resulted in recall bias. Furthermore, we could not include HDP or GDM history as some patients with childbearing histories forgot the details of their last obstetric experiences.

Overall, our study demonstrated that despite the increased risk of GDM, non-assisted hatching TE biopsy is not associated with the increased risks of HDPs, abnormal placentation, preterm birth, postpartum hemorrhage, and prolonged stay (both mothers and infants), compared with ICSI pregnancies. Despite the findings of our study, further study and validation need to be conducted.

## Data Availability Statement

The raw data supporting the conclusions of this article will be made available by the authors, without undue reservation.

## Ethics Statement

The studies involving human participants were reviewed and approved by the Ethics Committee of the Center for Reproductive Medicine, Shandong University. The patients/participants provided their written informed consent to participate in this study.

## Author Contributions

JY and Z-JC conceived and designed this study. SL conducted the data collection and analyses and wrote the manuscript. LC revised the manuscript and the other authors reviewed the manuscript. All authors were involved in interpreting the data and approved the final version.

## Funding

This study was generously funded by the Ministry of Science and Technology, PRC (2021YFC2700604, 2018YFC1002804).

## Conflict of Interest

The authors declare that the research was conducted in the absence of any commercial or financial relationships that could be construed as a potential conflict of interest.

## Publisher’s Note

All claims expressed in this article are solely those of the authors and do not necessarily represent those of their affiliated organizations, or those of the publisher, the editors and the reviewers. Any product that may be evaluated in this article, or claim that may be made by its manufacturer, is not guaranteed or endorsed by the publisher.
