# Supplementary material for: Non-Assisted Hatching Trophectoderm Biopsy Does Not Increase The Risks of Most Adverse Maternal and Neonatal Outcome and May Be More Practical for Busy Clinics: Evidence From China
Source: Front Endocrinol (Lausanne). 2022 Feb 17;13:819963. doi: 10.3389/fendo.2022.819963 (PMC8892202; doi:10.3389/fendo.2022.819963)
Supplement: Supplementary file 1 [file DataSheet_1.docx]

**Supplement Table 1** Odds ratios and 95% confidence intervals for maternal and neonatal outcomes by embryo biopsy status

|  | **Adjusted OR** | **95% CI** | **P-value** |
| --- | --- | --- | --- |
| PIH+Preeclampsia | 1.101 | 0.802--1.512 | 0.551 |
| Preeclampsia with severe features+Eclampsia | 1.495 | 0.623--3.589 | 0.368 |
| GDM* | 1.522 | 1.141--2.031 | 0.004 |
| HDP+GDM | 1.648 | 0.632--4.294 | 0.307 |
| Abnormal placentation | 1.410 | 0.777--2.558 | 0.259 |
| Umbilical cord abnormalities* | 11.539 | 1.199--111.067 | 0.034 |
| Abnormal amniotic fluid | 0.986 | 0.473--2.057 | 0.971 |
| Preterm birth | 1.103 | 0.834--1.458 | 0.491 |
| Cesarean section | 0.958 | 0.812--1.132 | 0.616 |
| Low birth weight | 1.381 | 0.983--1.939 | 0.063 |
| Postpartum hemorrhage | 1.922 | 0.578--6.390 | 0.287 |
| Prolonged stay for mothers | 1.528 | 0.895--2.610 | 0.120 |
| Prolonged stay for infants | 1.218 | 0.887--1.671 | 0.223 |

This Table corresponds to **Figure 3** in the article.

Adjusted for the maternal age, maternal BMI, times of previous miscarriages, parity, endometrial preparation protocols, endometrial thickness of transfer day, hCG ratio, PCOS, thyroid disorders, chronic hypertension, family history of hypertension, diabetes, family history of diabetes, history of uterine surgery, and neonatal sex. ICSI group is the reference group.

PIH, Pregnancy-induced hypertension; GDM, Gestational diabetes; HDP, Hypertensive disorders of pregnancy.

***** Statistically significant.

**Supplement Table 2** Comparisons of demographic characteristics between the non-assisted hatching biopsy group and matched ICSI group

|  | **Non-assisted hatching biopsy group (n=1088)** | **Matched ICSI group**  **(n=1086)** | **P-_value_** |
| --- | --- | --- | --- |
| Age (years) | 31 (3.5) | 32 (2.5) | 0.101^a^ |
| BMI (kg/m²) | 22.66 (2.23) | 22.77 (2.32) | 0.490^a^ |
| Parity | Primiparous: 72.24% (786) Multiparous: 27.76% (302) | Primiparous:68.60% (745) Multiparous: 31.40% (341) | 0.063^b^ |
| Neonatal weight (g) | 3500 (300) | 3500 (300) | 0.050^a^ |
| Neonatal sex ratio | Female: 46.78% (509)  Male: 53.22% (579) | Female: 43.46% (472)  Male: 56.54% (614) | 0.120^b^ |

Data are shown as Median (Quartile deviation, QD), and (%) (Number of positive cases).

**^a^** Mann-Whitney U Test.

**^b^** Pearson's chi-Squared Test.

ICSI, Intracytoplasmic sperm injection; BMI, Body mass index.

**Supplement Table 3** Odds ratios and 95% confidence intervals for maternal and neonatal outcomes after matching

|  | **Adjusted OR** | **95% CI** | **P-value** |
| --- | --- | --- | --- |
| PIH+Preeclampsia | 1.61 | 0.831--3.121 | 0.158 |
| Preeclampsia with severe features+Eclampsia | 3.55 | 0.48--26.234 | 0.214 |
| GDM | 1.145 | 0.589--2.227 | 0.689 |
| HDP+GDM | 1.095 | 0.095--12.59 | 0.942 |
| Abnormal placentation | 3.241 | 0.943--11.144 | 0.062 |
| Abnormal amniotic fluid | 1.244 | 0.27--5.743 | 0.779 |
| Preterm birth | 0.851 | 0.397--1.824 | 0.679 |
| Cesarean section | 0.757 | 0.537--1.068 | 0.113 |
| Low birth weight | 1.225 | 0.451--3.328 | 0.691 |
| Prolonged stay for mothers | 0.812 | 0.224--2.947 | 0.752 |
| Prolonged stay for infants | 1.665 | 0.817--3.391 | 0.160 |

This Table corresponds to **Figure 4** in the article.

Adjusted for the maternal age, maternal BMI, times of previous miscarriages, parity, endometrial preparation protocols, endometrial thickness of transfer day, hCG ratio, PCOS, thyroid disorders, chronic hypertension, family history of hypertension, diabetes, family history of diabetes, and history of uterine surgery. Matched ICSI group is the reference group.

PIH, Pregnancy-induced hypertension; GDM, Gestational diabetes; HDP, Hypertensive disorders of pregnancy.

**Supplement
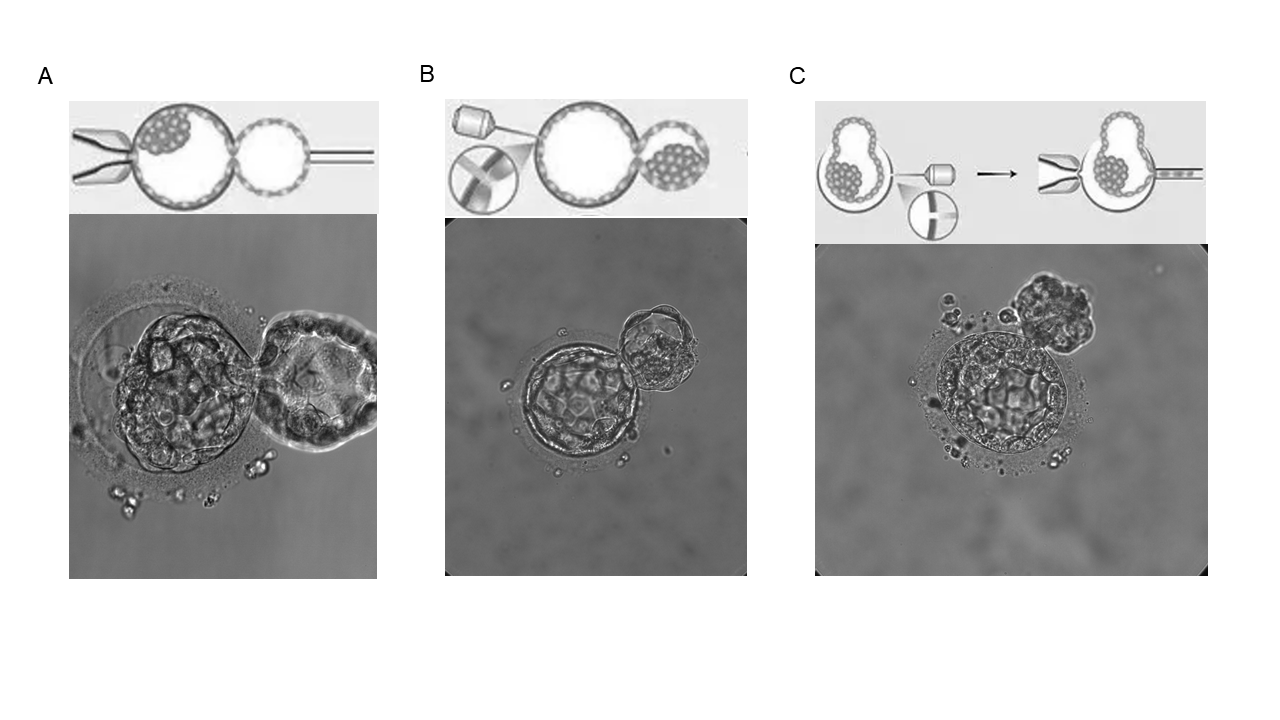
 Figure 1 |** Several other non-assisted hatching biopsy strategies. **(A)** After laser drilling, directly remove the trophoblast cells outside zone pellucida by pulling and suction; **(B)** Using the laser to open one side of the zone pellucida to draw out the inner cell mass, then drilling the other side with the laser to aspirate the trophoblast cells; **(C)** Laser was used to opening the zona pellucida near the inner cell mass, then the entire blastocyst was positioned by pulling the herniated trophectoderm, the zona pellucida on the opposite side of the inner cell mass was opened with the laser to harvest trophoblast cells.


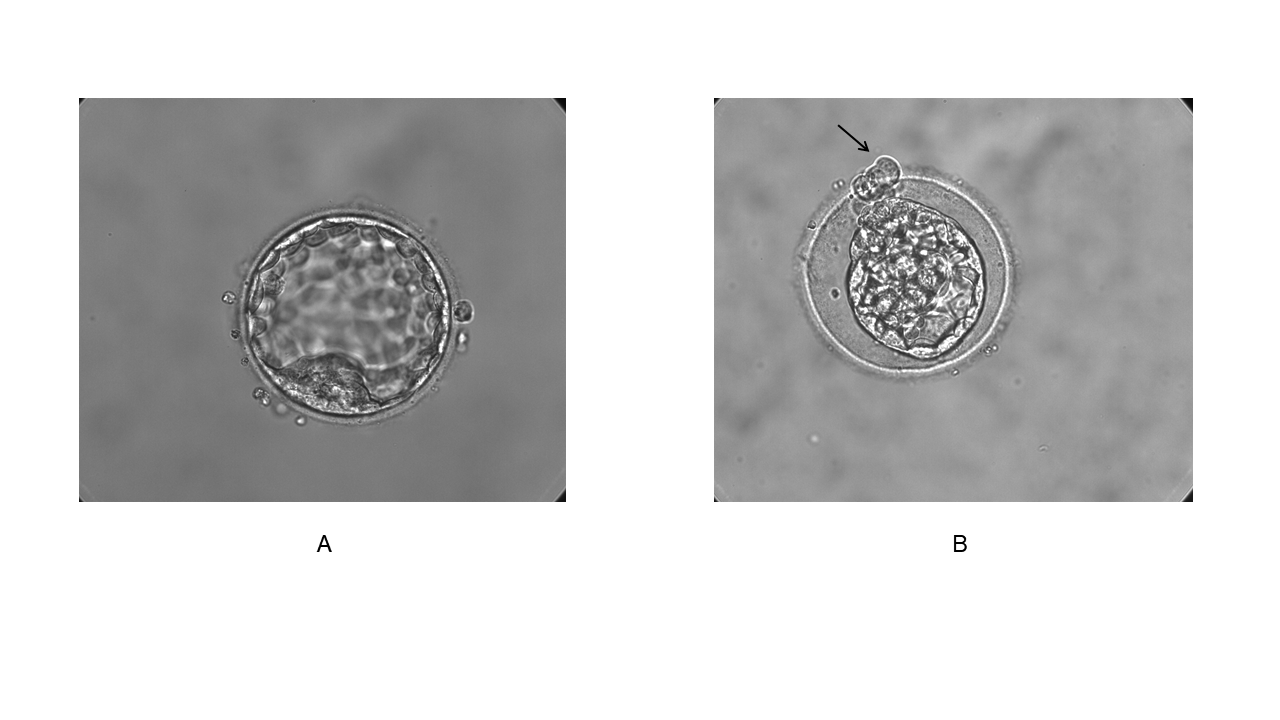


**Supplementary Figure 2 |** The pre-assisted hatching embryo did not fully expand as much as the non-assisted hatching embryo. **(A)** A stage 4 blastocyst on day 5; **(B)** A day 5 blastocyst after day-3 hatching. Here, some cellular components of the blastocyst had herniated through the defect on zone pellucida, but it had not met the standards of stage 4 blastocyst.
